# Reversible Cerebral Vasoconstriction Syndrome: An Important Cause of Acute Severe Headache

**DOI:** 10.1155/2012/303152

**Published:** 2012-07-09

**Authors:** Li Huey Tan, Oliver Flower

**Affiliations:** Intensive Care Medicine, Royal North Shore Hospital, St. Leonards, NSW 2065, Australia

## Abstract

Reversible cerebral vasoconstriction syndrome (RCVS) is an increasingly recognized and important cause of acute headache. The majority of these patients develop potentially serious neurological complications. Rigorous investigation is required to exclude other significant differential diagnoses. Differentiating RCVS from subarachnoid haemorrhage (SAH) and primary angiitis of the central nervous system (PACNS) may be difficult but has important therapeutic implications. This paper describes what is currently known about the epidemiology, pathophysiology, clinical, and diagnostic features of the syndrome, an approach to investigation, a summary of treatments, and what is known of prognosis.

## 1. Introduction

Acute severe headache presenting to the Emergency Department (ED) accounts for 1-2% of admissions [1]. Whilst the differential diagnosis in the setting of nontraumatic headache is extensive, it is imperative that life-threatening causes of headache are identified in a timely fashion and treated appropriately. Reversible cerebral vasoconstriction syndrome (RCVS) is one of these differentials that potentially has dire consequences and, with improving technology and awareness, is being increasingly diagnosed. 

The presence of acute severe headache and characteristic angiographic findings was initially described in a case series in which Gregory Call and Marie Fleming were lead authors, hence the eponym Call-Fleming syndrome [2]. They described unique features in patients who presented with sudden onset severe headache and cerebral angiography that demonstrated reversibility of vasoconstriction of arteries involving the Circle of Willis and its immediate branches [2]. Other literature has described similar clinical entities that appear to fall under the descriptive heading of RCVS. This includes migrainous vasospasm or migraine angiitis [3–5], benign angiopathy of the central nervous system [6], postpartum angiopathy [7], thunderclap headache with reversible vasospasm [3–5], and drug-induced angiopathy [7, 8]. Distinguishing all of these disorders from cerebral vasculitis has also been challenging but is a key diagnostic step as the treatments are significantly different. The unifying term reversible cerebral vasoconstriction syndrome (RCVS) was proposed by Calabrese in 2007 [9]. It encompasses all of these clinical entities, which share similar clinical presentations, radiological findings, and sequelae. The diagnostic elements of RCVS are shown in Table 1.

Since the initial descriptions in 1988, much remains unknown about RCVS and this is reflected in the paucity of literature on the topic. This is partially due to a previous lack of a consensus definition, deficits in understanding of the underlying pathophysiology, and overlapping features with other conditions such as cerebral vasculitis. The incidence of RCVS appears to be increasing. This may be due to the increasing availability and advances in neurovascular imaging, or a genuine increase, perhaps related to more prevalent use of vasoactive substances [10].

## 2. Epidemiology

There is a clear female predominance of RCVS in all published case series, with female to male ratios ranging from 2.6 : 1 [11] to 10 : 1 [12]. These differences may be due to geographical and genetic reasons. Sex predilection seems to be less significant in secondary RCVS [11]. The typical age group affected in adulthood is between 20 and 50 years old. However, there have been case reports of patients under 18 years of age, the majority being male [13, 14].

RCVS can occur spontaneously or be secondary to a precipitating factor. The proportion of spontaneous cases has varied depending on the population studied, from 37% in a French study [14] to 96% in a Taiwanese cohort [12]. Vasoactive drugs and the postpartum period are two common associations [14], with several other associations being suggested from previous case series (see Table 2).

## 3. Pathophysiology

The pathogenesis of RCVS remains poorly understood. Current consensus on the aetiology focuses around alteration of cerebral vascular tone. This may occur spontaneously (primary RCVS) or be triggered by endogenous or exogenous substances (secondary RCVS) (see Table 2). There appears to be interaction between sympathetic overactivity and endothelial dysfunction, resulting in dysautoregulation [11]. With the radiological similarities with postsubarachnoid haemorrhage vasospasm, it has been postulated that the mediators of vasospasm in subarachnoid haemorrhage such as endothelin-1, serotonin, nitric oxide, prostaglandins, and catecholamines [15, 16] may also be invoked in RCVS by different mechanisms. It has also been suggested that a sudden central neuronal discharge may induce vasospasm and the severe headache be caused by stimulation of the sensory afferents of the first division of the trigeminal nerve and dorsal root of C2 which supply these cerebral blood vessels [9]. Resolution of symptoms does not always correlate with radiological resolution of vasoconstriction, and the factors perpetuating this process are also yet to be identified. Genetic factors are likely to play a role in the predisposition and development of RCVS.

Postpartum angiopathy is considered a variant of RCVS occurring after pregnancy. It can occur following uncomplicated pregnancy as well as in eclampsia [17]. Acute severe headaches tend to occur within days or weeks after uncomplicated deliveries unlike those seen in eclampsia. The imbalance of angiogenic factors seen in eclamptic patients has not been demonstrated in patients with uncomplicated pregnancy that develop postpartum angiopathy.

## 4. Clinical Features

The most common symptom of RCVS is an acute severe “thunderclap” headache (TCH), typically in females between the ages of 20 and 50 years, and this is often the only symptom at presentation [14]. This TCH is defined as a severe headache reaching its maximal intensity within one minute [10]. The headache tends to be recurrent, over a period of days to weeks. Characteristics of the headache vary widely from occipital to diffuse and constant to throbbing. It may occur spontaneously or be precipitated by exercise or a valsalva manoeuvre. Systemic clinical features such as nausea, vomiting, and transient hypertension are not uncommon.

Neurological deficits may or may not be present initially. In a recent cohort study from North America, focal neurological deficits were present initially in 43% [14]. These deficits include visual disturbances, photophobia, blindness, focal facial or limb weakness, dysarthria, and ataxia. Generalised tonic-clonic seizures occurred in 17%. Most significantly, severe and permanent neurological deficits and even death may occur as a consequence. The reversible component suggested in the description refers specifically to the angiographic vasoconstriction, and the feared vascular complications are surprisingly common (81%). These include ischaemic stroke, nonaneurysmal subarachnoid haemorrhage, intracerebral haemorrhage, cerebral oedema and posterior reversible leucoencephalopathy syndrome (PRES). Most of these complications occur in the first week of presentation, except cerebral ischaemia, which is more common in and after the second week [18]. Female gender and a history of migraines are both independent risk factors for intracranial haemorrhage [19]. Therefore, RCVS should be considered in patients presenting with cryptogenic stroke, particularly if there was an associated typical headache and imaging reveals symmetrical brain infarctions and oedema.

## 5. Investigations

**Figure 1:**
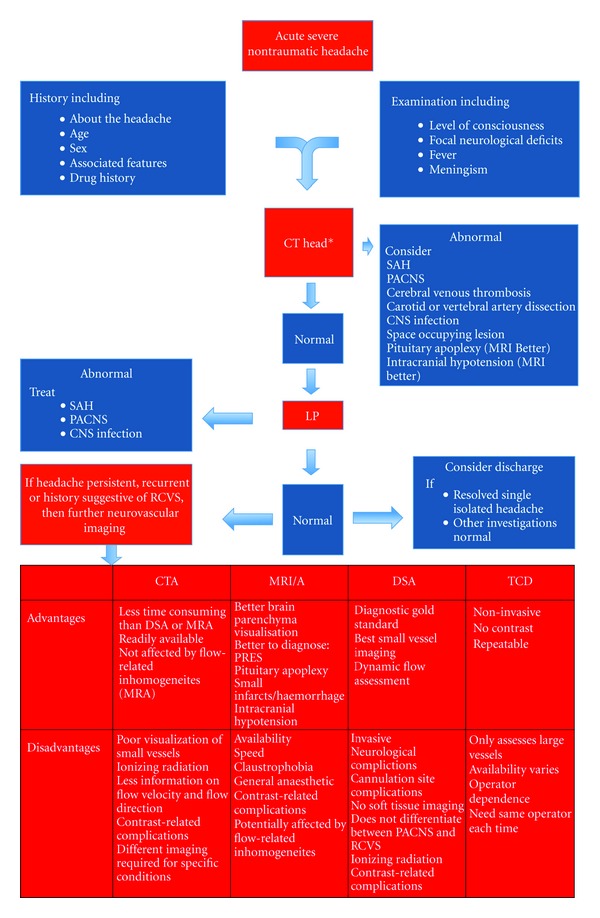
An approach to investigation of RCVS [10]. *CT angiography may be considered at this stage, specifically looking for cervical artery dissection, cerebral venous thrombosis or RCVS, depending on the history, clinical suspicion and contraindications to radiocontrast.

An approach to investigation of acute severe nontraumatic headache is outlined in Figure 1. A noncontrast CT brain should be performed initially to exclude subarachnoid and intracerebral haemorrhage. If normal, this should be followed by a lumbar puncture (LP). In the majority of acute presentations of RCVS, the noncontrast CT head shows no abnormalities [14]. Depending on the history, CT angiography at the time of a non-contrast CT may be warranted, looking for evidence of RCVS, cervical artery dissection, or cerebral venous thrombosis. It should be noted that all of these diagnoses require different CT imaging techniques and this should be discussed with the radiologist and radiographer involved to obtain optimal images. Other differentials to consider include pituitary apoplexy and intracranial hypotension. These both have characteristic CT findings but are better visualised with an MRI, which may follow if the history and examination are suggestive and the CT is nondiagnostic. The LP is performed looking for evidence of CNS infection, subarachnoid haemorrhage, or primary angiitis of central nervous system (PACNS). A distinguishing feature of RCVS is an initially normal CSF result. After a single episode of an acute non-traumatic headache that has resolved, if the CT with contrast and the LP are normal, it may be reasonable to consider discharge if they can be relied upon to return if their symptoms reoccur.

For persistent or recurrent acute severe headaches, four different imaging modalities are currently used to evaluate the presence of vasospasm, summarized in Figure 1. In general, an approach starting with less invasive imaging is employed. Angiographic changes in cerebral arteries, described as a “string of beads,” are highly characteristic of RCVS.

CT angiography (CTA) is readily available, fast, and can be performed immediately after an initial non-contrast CT. It is not affected by flow-related inhomogeneities that can affect MRI and can certainly reveal regions of vasospasm. CT venography can also exclude cerebral sinus thrombosis, an important differential diagnosis. However, CTA may lack the sensitivity of digital subtraction cerebral angiography (DSA), may poorly visualise smaller distal vessels, has no scope for intervention, and incurs contrast and radiation exposure. Modern multidetector-row spiral CT angiography produces vascular imaging potentially equivalent to DSA [20], unlike older generations of CT scanners. When looking for evidence of RCVS with a CTA, the images must include all the cerebral arteries up to the vertex, so as not to miss spasm in these vessels, which must also be considered when arranging the imaging.

MRI with angiography and venography has advantages over CTA as the next radiological investigation following a normal CT [10] and has been validated in this context [21]. The MR sequences should as a minimum include T1, T2, fluid attenuated inversion recovery imaging, gradient-echo (T2) imaging, diffusion weighted imaging, and apparent diffusion coefficient mapping for differential diagnosis and evaluation of complications. Cervical MR using a T1 fat-saturation sequence with contrast should be considered if cervical artery dissection is suspected [21]. MRA avoids potential complications of repeated DSA's, and the improved soft tissue imaging may demonstrate small areas of cortical haemorrhage, ischaemic complications of RCVS not visible with CT, or changes consistent with PRES. However, MRA still lacks the sensitivity for vascular imaging of DSA, and imaging small arteries in the setting of PACNS is more difficult with MRA [22]. MRA is also not always immediately available and potentially carries the risks of transport, remote-site anaesthesia and the complications of the gadolinium contrast.

Currently, DSA is still considered to be the gold standard for the diagnosis of RCVS. It allows real-time appreciation of vessel calibre and flow and permits better visualization of the smaller, peripheral vessels with superior spatial and temporal resolution. More significantly, there is also the potential for intervention with intra-arterial vasodilators in addition to the diagnostic advantages. The disadvantages of DSA include the invasiveness, potential vascular injury with stroke, and the inherent radiation and contrast exposure. One case series reported a high incidence (9%) of transient neurological deficit post-DSA in patients with RCVS [11] however, this was likely to be related to the underlying pathology, and rates of 0.5% for permanent and 1% for transient neurological complications may be expected [23–25].

Transcranial doppler (TCD) imaging has a potential role in monitoring vasospasm after RCVS has been diagnosed by another imaging modality. It is a noninvasive way to assess larger vessel vasospasm, and in one study of TCD in RCVS, a mean flow velocity of the middle cerebral artery greater than 120 m/s was associated with a greater risk of ischaemic complications [26]. However, TCD does not allow assessment of smaller vessels, is not always available, may be technically difficult on some individuals, and is subject to significant inter-observer and interindividual variability. Centres where there is local expertise and the same operator is available to repeat the imaging on a regular basis may use serial TCD to avoid the risks of the other imaging techniques. Cervical artery dopplers have been used to investigate for arterial dissection in the context of acute severe headache; whilst having a significantly favourable complication profile compared to any form of angiogram, there are bony regions limiting ultrasound imaging, greatly reducing the sensitivity of this investigation.

None of these imaging modalities give an irrefutable diagnosis of RCVS. The choice of imaging modality should be chosen based upon the other differential diagnoses suggested from the history, and what is available. A CTA or MRA may follow a non-contrast CT however, if both of these are nondiagnostic and clinical suspicion of RCVS exists, a DSA should follow. Follow-up imaging may be with MRA or DSA if intra-arterial interventions are being considered.  Table 3 shows comparative features of RCVS, cervical artery dissection, PACNS, and SAH to aid in diagnosis. Angiographically, SAH-induced vasospasm is more commonly longsegmental and mainly around the bleeding focus [27], compared to the multiple, short-segmental and diffuse changes seen in RCVS; however, this is not 100% specific and a DSA is also required to look for evidence of an aneurysm if this has not already been identified. PACNS is radiologically identical to RCVS, making the clinical history, risk, factors and CSF studies important in differentiating these conditions. The reversibility of the angiographic findings is a key component to the diagnosis but only helpful retrospectively. Therefore, if a patient presents with a classic history of repetitive thunderclap headaches, has no evidence of SAH, has normal CSF analysis and a normal MRI, and shows the typical findings of RCVS on vascular imaging (DSA, CTA, or MRA), a diagnosis of RCVS can be made. If the history or CSF analysis is ambiguous, then the diagnosis of PACNS must be entertained.

**Figure 2:**
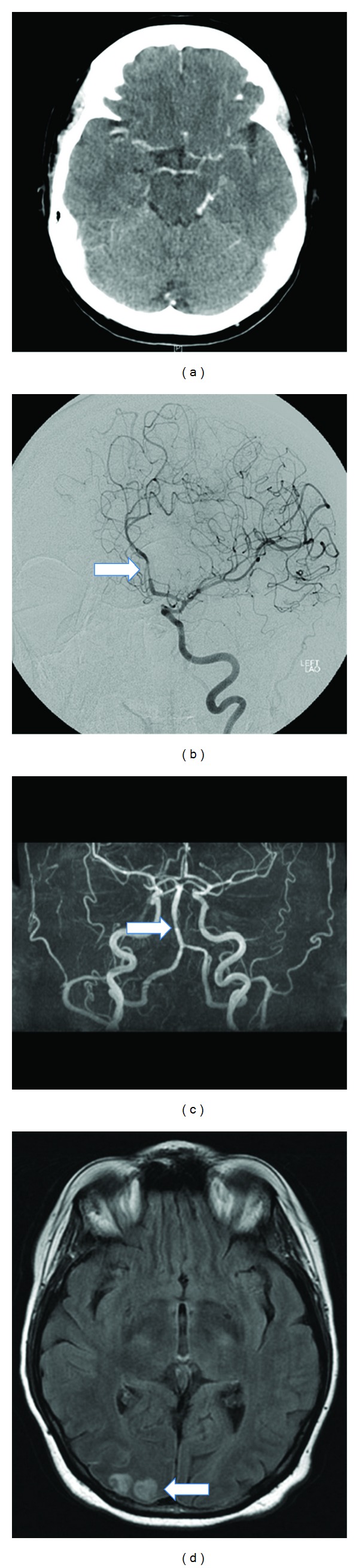
Neuroimaging in a case of RCVS. Neuroimaging of a 61-year-old female with RCVS. (a) CT angiography demonstrated no evidence of vasospasm. (b) DSA demonstrated diffuse areas of focal segmental narrowing affecting both the anterior and posterior circulation, particularly in the A2 segment of the left anterior cerebral artery (arrow). (c) MRA showed predominantly peripheral focal segmental spasm, though not as clearly as the DSA (d) MRI 6 weeks after presentation reveals high T2 signal representing right occipital cortical infarcts. CT: computerised tomography; DSA: digital subtraction angiogram; MRA: magnetic resonance angiogram; MRI: magnetic resonance imaging.

Figure 2 shows some neuroimaging of a 61-year-old lady with RCVS. These images illustrate the limitations of CTA in detecting peripheral vasospasm, the benefits of DSA imaging (which was obtained whilst intra-arterial verapamil was administered), the limitations of MRA compared to DSA, and the infarctions which can develop as a complication of vasospasm.

## 6. Treatment

The evidence for effective treatment in RCVS is limited to observational studies at present. Any potential drugs or triggers should be discontinued or avoided in secondary RCVS [9, 10, 14, 18]. Glucocorticoids were previously considered a potential treatment; however, they have more recently been shown to be an independent predictor of poor outcome [14] and should be avoided. This highlights the importance of distinguishing the two entities as the use of steroids (prednisolone 1 mg/kg/day) is the treatment of choice in PACNS [28].

The calcium channel blocker nimodipine is the most widely employed treatment for RCVS, although there are no prospective randomised placebocontrolled trials to support this. Nimodipine has been shown to terminate the headache in 64–83% of patients [11, 12, 29], although in the largest case series reported, treatment with nimodipine showed no outcome benefit over symptomatic treatment alone [14]. As in SAH, both oral and intravenous nimodipine regimens have been used and there is no published evidence supporting one over the other.

Other systemic treatments that have been used include intravenous and oral nicardipine [13], intravenous and oral verapamil [30], and intravenous magnesium sulphate (in the treatment of postpartum angiopathy) [31]. These reports all have the inherent limitations of case studies. Intra-arterial (IA) vasodilators injected during DSA with and without angioplasty are also used. These include IA milrinone [30], IA verapamil [32], and IA nimodipine as both a therapeutic and diagnostic agent [33], with the evidence, again, limited to case reports. IA verapamil has been shown to improve radiological vasospasm [34, 35], but whether this translates to improved clinical outcomes remains to be proven. With the current agnosticism regarding optimal treatment, multicentre, prospective, randomized, placebocontrolled trials would seem prudent, though logistically difficult.

## 7. Prognosis

The most serious complications of RCVS are permanent neurological deficit and death. In the largest North American study, 81% of patients developed radiological evidence of brain lesions as a consequence of RCVS 39% had ischemic infarcts, 34% had convexity subarachnoid haemorrhage, 20% developed lobar intracerebral haemorrhage, and 38% had cerebral edema [14]. Despite this, the rate of permanent neurological disability is surprisingly low. In this cohort, 89% had a good clinical outcome (Modified Rankin Score at followup or discharge of 0–3) [14], and in a systematic review, 71% had no evidence of any long-term disability, 29% had only minor disability [18], and 6% had permanent neurological disability [11]. Cerebral infarction and intracerebral haemorrhage are predictors of a worse outcome [14]. Deaths from RCVS have been reported in the literature but are rare [14, 36]. The rate of recurrence is approximately 8% [21].

## 8. Conclusion

RCVS is a clinical entity and neurological emergency that is being diagnosed with increasing frequency but is still underrecognized, and a high index of suspicion is essential. There are characteristic features in the history and on neuroimaging that are distinctive, but overlapping features with other conditions can make diagnosis difficult. Distinguishing RCVS from PACNS is important, as the glucocorticoid treatment indicated for PACNS appears to be harmful in RCVS. The management is predominantly supportive, whilst ruling out other life-threatening neurological conditions, identifying risk factors, and discontinuing offending agents. Intra-arterial vasodilators and balloon angioplasty offer promise but as yet have not been proven to improve clinical outcomes. There are still many areas for future research including the pathogenesis, the natural history of the syndrome, the optimal diagnostic strategy and the treatment. 

## Figures and Tables

**Table 1:** Diagnostic criteria for RCVS [9].

Summary of critical elements for the diagnosis of reversible cerebral vasoconstriction syndromes
(1) Angiography (DSA, CTA, or MRA) documenting multifocal segmental cerebral artery vasoconstriction	
(2) No evidence of aneurysmal subarachnoid hemorrhage	
(3) Normal or near-normal cerebrospinal fluid analysis (protein level <80 mg%, leukocytes <10 mm^3^, normal glucose level)	
(4) Severe, acute headaches, with or without additional neurologic signs or symptoms.	
(5) Reversibility of angiographic abnormalities within 12 weeks of symptom onset. If death occurs before the follow-up studies are completed, autopsy rules out such conditions as vasculitis, intracranial atherosclerosis, and aneurysmal subarachnoid hemorrhage, which can also manifest with headache and stroke	

**Table 2:** Secondary precipitants of RCVS [9–12].

Vasoactive substances	Predisposing conditions
Recreational drugs: *Cannabis*, cocaine, ecstasy, amphetamines, LSD, binge drinking	Pregnancy
Sympathomimetics, nasal decongestants: ephedrine, pseudoephedrine	Eclampsia, preeclampsia
Serotonergic drugs: selective serotonin reuptake inhibitors, triptans	Neoplasia: phaeochromocytoma, bronchial carcinoid, glomus tumour
Immunosuppressants: tacrolimus, cyclophosphamide	Neurosurgery, head injury
Nicotine patches	Hypercalcaemia
Herbal medications: ginseng	Porphyria
Blood products: erythropoietin, immunoglobulin, red cell transfusion	Intracerebral haemorrhage, subarachnoid haemorrhage

**Table 3:** Distinguishing features of RCVS, cervical artery dissection, PACNS and SAH [9].

	RCVS	Cervical artery dissection	PACNS	SAH
History	Sudden onset headache, often thunderclap	Sudden or subacute, can have thunderclap features	Insidious, constant, progressive, dull	Sudden onset headache, often thunderclap
	More common in females	No sex predilection	No sex predilection	More common in females
	Age 20–50 years old	Age less than 50 years old	Age 40–60 years old	Age 40–60 years old
				Risk increases with age
				Likely to be younger in familial SAH

Risk factors	Drugs, pregnancy, tumours, neuro injury, idiopathic	Atherosclerosis, cervical trauma, connective tissue disease. Can be idiopathic		Family history Known cerebral aneurysm

Examination	Presence or absence of neurological deficit	Presence or absence of neurological deficit. Important to rule out in younger patients.	Presence or absence of neurological deficit, 5% spinal involvement	Depends on severity of haemorrhage

CT brain	Majority normal Cortical SAH, ICH	Normal in the absence of cerebral infarct (60%); crescenteric intramural haematoma on CTA	Majority abnormal—diffuse, multiple small infarcts	Majority abnormal. SAH, cerebral oedema, hydrocephalus

CSF studies	Majority normal	Normal	Majority abnormal—raised protein, cell count	Abnormal—xanthochromia, raised red cell count

MRI brain	Majority normal	MRA may reveal intramural haematoma as well as demonstrate flow abnormalities. More sensitive than CT or early infarction	Nonspecific changes Multifocal, cortical or subcortical infarcts, diffuse white matter changes, or leptomeningeal enhancement	Areas of infarct corresponding to vascular territory involved

Cerebral angiography	Considered gold standard. Useful in recurrent TCH Diffuse segmental stenosis—medium, large arteries	Long-segmental stenosis, intimal flaps, arterial pseudoaneurysm	Unable to visualise changes in small arteries	Aneurysm, arterio-venous malformation Vasospasm (not multifocal) at Day 4

CNS biopsy	Not indicated		Gold standard. Skip, segmental vascular lesions	
